# Amelioration of Experimental Autoimmune Encephalomyelitis by Plumbagin through Down-Regulation of JAK-STAT and NF-κB Signaling Pathways

**DOI:** 10.1371/journal.pone.0027006

**Published:** 2011-10-31

**Authors:** Yan Jia, Ji Jing, Yang Bai, Zhen Li, Lande Liu, Jian Luo, Mingyao Liu, Huaqing Chen

**Affiliations:** 1 Institute of Biomedical Sciences and School of Life Sciences, East China Normal University, Shanghai, China; 2 Department of Molecular and Cellular Medicine, Institute of Biosciences and Technology, Texas A&M University Health Science Center, Houston, Texas, United States of America; Emory University, United States of America

## Abstract

Plumbagin(PL), a herbal compound derived from roots of the medicinal plant *Plumbago zeylanica*, has been shown to have immunosuppressive properties. Present report describes that PL is a potent novel agent in control of encephalitogenic T cell responses and amelioration of mouse experimental autoimmune encephalomyelitis (EAE), through down-regulation of JAK-STAT pathway. PL was found to selectively inhibit IFN-γ and IL-17 production by CD4^+^ T cells, which was mediated through abrogated phosphorylation of JAK1 and JAK2. Consistent with IFN-γ and IL-17 reduction was suppressed STAT1/STAT4/T-bet pathway which is critical for Th1 differentiation, as well as STAT3/ROR pathway which is essential for Th17 differentiation. In addition, PL suppressed pro-inflammatory molecules such as iNOS, IFN-γ and IL-6, accompanied by inhibition of IκB degradation as well as NF-κB phosphorylation. These data give new insight into the novel immune regulatory mechanism of PL and highlight the great value of this kind of herb compounds in probing the complex cytokine signaling network and novel therapeutic targets for autoimmune diseases.

## Introduction

As a chronic human autoimmune disease, multiple sclerosis (MS) is mainly initiated by inflammatory responses of T lymphocytes reactive to myelin antigens in the central nervous system (CNS), leading to inflammatory demyelination and subsequent axonal injury [1], [2]. The animal model, experimental autoimmune encephalomyelitis (EAE), is widely used to unravel the mechanisms of autoimmune inflammation in MS [3]. It is well known that autoreactive T helper (Th) cells are involved in the disease pathogenesis. Th1 cells (IFN-γ producing CD4^+^ T cells) and Th17 cells (IL-17 producing CD4^+^ T cells) play critical roles in the effector stage of EAE, whereas Th2 cells and Th2-type cytokines such as IL-4, IL-10 downregulate the disease [4]. EAE is markedly suppressed in mice lacking IL-17 or IL-17 receptor, and IL-17-specific inhibition attenuates inflammation [5]–[9]. Through production of proinflammatory cytokines such as IL-6, IFN-γ, IL-17, Th1 and Th17 cells recruit other inflammatory cells into CNS lesions, resulting in chronic inflammation and demyelination of CNS [4], [10].

It is understood that the JAK-STAT pathway is the major signaling pathway regulating Th1 and Th17 differentiation and their functions [11]. Among STATs which mainly activated through phosphorylation by JAK1, JAK2 and TYK2, STAT1 and STAT4 are involved in Th1 differentiation while STAT3 is activated by IL-6 and IL-23 and mainly involved in Th17 differentiation [12], [13]. These are mediated through key transcription factors such as T-bet and RORγt/RORα, each for Th1 and Th17 specifically [14]–[16]. There are cross regulations between STAT and NF-κB signaling pathways. Besides, as cytokines and NF-κB signaling pathways are deeply related cascades that involved in immune response and inflammation, NF-κB regulates the expression of many proinflammatory cytokines such as IFN-γ, IL-6 [17].

In a recent study, the principal MS therapies in current clinical use were extensively analyzed, including IFN-β and glatiramer acetate [18]. However, these are only modestly effective, or have certain safety concerns. In this regard, exploiting novel anti-inflammatory compounds that can target multiple checkpoints or mechanisms is certainly a priority.

Plumbagin (5-hydroxy-2-methyl-1,4-naphthoquinone, PL) is a natural bicyclic naphthoquinone found in the plants of *Droseraceae*, *Plumbaginaceae*, *Ancistrocladaceae* and *Dioncophyllaceae* families. It has many potent biological activities, including antioxidant [19], [20], anti-inflammatory [21]–[24], anti-tumor [22], [25], antibacterial and antifungal activities [26], [27]. PL exhibits its anti-tumor activity mainly through suppressing NF-κB signal pathway [28]. As a critical transcription factor, NF-κB also has its central role in regulating leukocytes proliferation, expression of immunoregulatory genes and adaptive immune responses. It is found that PL inhibited T cell proliferation in response to polyclonal mitogen Concanavalin A (Con A) by blocking cell cycle progression and accompanied by a decrease in the level of Con A-induced IL-2, IL-6 and IFN-γ [21].

Based on above observations, we hypothesized that the immunomodulatory effects of PL may be exploited for use in the treatment of autoimmune disorders. In this study, we investigated the potential regulatory properties of PL and its underlying mechanism in mouse EAE. Results presented here provide new insight into the novel regulatory mechanism of herbal compounds such as PL in treatment of autoimmune diseases.

## Materials and Methods

### Mice

Female C57BL/6 (B6) mice were purchased from the Shanghai Laboratory Animal Center, Chinese Academy of Sciences, Shanghai. Mice were maintained under pathogen-free conditions. Plumbagin (with purity greater than 97%) was purchased from Sigma-Aldrich. A 100 mM solution of plumbagin was prepared in DMSO, stored at −20°C.

### Induction and treatment of EAE

For EAE induction, B6 mice (10–12 wk) were immunized s.c. with 300 µg of myelin oligodendrocyte glycoprotein (MOG residues 35–55). Sequence of the peptide was Met-Glu-Val-Gly-Trp-Tyr-Arg-Ser-Pro-Phe-Ser-Arg-Val-Val-His-Leu-Tyr-Arg- Asn-Gly-Lys and displayed a purity of >95% (GL Biochem). Immunization was performed by mixing MOG peptide in Complete Freund's Adjuvant containing 5 mg/ml heat-killed H37Ra, strain of Mycobacterium tuberculosis (Difco Laboratories). 400 ng Pertussis toxin (List Biological Laboratories) in PBS-50 mM NaCl was administered i.p. on the day of immunization and 24 h later. For treatment of EAE, PL was administered at 2 mg/kg or DMSO (Sigma-Aldrich) as vehicle control i.p. daily from day 7 postimmunization onwards. The prevention protocol differed from the treatment protocol only at the start of PL administration (3 days before immunization). Mice were weighed and examined daily for disease symptoms, assessed using the standard score system: 0, No obvious changes in motor functions; 1.0, Limp tail; 2.0, Limp tail and wobbly gait; 3.0, Bilateral hind limb paralysis; 4.0, Complete hind limb and partial fore limb paralysis; 5.0 Death. Animal procedures were approved by the institutional Animal Ethics Committee of East China Normal University.

### Histopathology

After intracardiac fixative perfusion, spinal cords were isolated from mice, transcardially perfused with 4% paraformaldehyde for overnight. Tissues were treated in ethanol and xylene as usual and paraffin-embedded 5 µm sections were stained with H&E or Luxol fast blue. For immunohistochemisty staining of CD4^+^ T cells, spinal cords were removed from mice, perfused with PBS, and incubated in 30% sucrose at 4°C overnight. Frozen specimens were sectioned at 5 µm with a cryostat. The frozen slices were developed with Histostain-Plus Kit (MR Biotech), according to the manufacturer's instructions.

### MOG-specific T cell proliferation assay

For *in vitro* proliferation, splenocytes were isolated from EAE mice and cultivated in triplicates in complete RPMI1640 medium (GIBCO22400, with 10% FBS) at 2×10^5^ per well in 96-well plates in presence or absence of the MOG peptide (20 µg/ml). PL was added *in vitro* at the indicated concentrations and all cultures were maintained at 37°C in 5% CO_2_ for 48 h. T cell proliferation was measured using a MTS kit (CellTiter 96® AQueous One Solution Cell Proliferation Assay, Promega), according to the manufacturer's instructions. For *ex vivo* assay, splenocytes were isolated 18 days postimmunization from DMSO vehicle-treated and PL-treated mice, and examined for proliferation in the presence or absence of MOG peptide.

### RT-PCR and quantitative real-time PCR analysis

Total RNA was extracted from splenocytes using TRIzol (Invitrogen). First-strand cDNA was synthesized using PrimeScriptTM RT reagent kit (Takara), according to the manufacturer's instructions. mRNA expression was determined by RT-PCR or quantitative real-time PCR. RT-PCR products were analyzed on a 1.5% multiwelled agarose gel. Electrophoresis was carried out in 1×TAE buffer at 100 V for 30 minutes. Real-time PCR was performed using SYBR Green Master Mix under standard thermocycler conditions. Data were collected and quantitatively analyzed on a Mx3005p quantitative PCR system (Stratagene). Results were presented as fold increases relative to the expression of house keeping β-actin. Sequences of PCR primers were listed in Table 1.

**Table 1:** Specific primers used in PCR analysis.

Molecule	Primer	Sequence
**β-actin**	Forward	5′-GCTGTCCCTGTATGCCTCT-3′
	Reverse	5′-GTCACGCACGATTTCCCTC-3′
**IL-13**	Forward	5′-GCTTATTGAGGAGCTGAG-3′
	Reverse	5′-GGGCTACTTCGATTTTGG-3′
**IL-4**	Forward	5′-TTTGAACGAGGTCACAGG-3′
	Reverse	5′-GCATGATGCTCTTTAGGC-3′
**IL-6**	Forward	5′-TTCTTGGGACTGATGCTG-3′
	Reverse	5′-CTGGCTTTGTCTTTCTTGTT-3′
**IL-17a**	Forward	5′-CTCAACCGTTCCACGTCAC-3′
	Reverse	5′-ACACCCACCAGCATCTTCT-3′
**IFN-γ**	Forward	5′-TCTGAGACAATGAACGCTAC-3′
	Reverse	5′-TGGACCACTCGGATGAG-3′
**iNOS**	Forward	5′-GGACGAGACGGATAGGCA-3′
	Reverse	5′-CATCTCGGGTGCGGTAG-3′
**RORα**	Forward	5′-CGCTCGTGGCTTCAGGAAAAGGT-3′
	Reverse	5′-AGAAGTGCTCGGGCGCGACAT-3′
**RORγt**	Forward	5′-CATCTCTGCAAGACTCATCG-3′
	Reverse	5′-CAGGGGATTCAACATCAGTG-3′
**TNFα**	Forward	5′-TCCCTTTCACTCACTGGC-3′
	Reverse	5′-ACTTGGTGGTTTGCTACG-3′
**Foxp3**	Forward	5′-AGGAGAAAGCGGATACC-3′
	Reverse	5′-CAGGGAGGAGTTCAGTAGAG-3′

### Measurement of cytokine production

At 18 days after MOG immunization, splenocytes at 2×10^6^ /ml isolated from PL-treated or vehicle-treated mice were stimulated with the MOG peptide (20 µg/ml) in complete RPMI 1640 medium. Supernatants were harvested after 48 h and assays for IFN-γ and IL-17 were performed in triplicates by ELISA using commercial kits (Dakewe Systems) according to the manufacturer's recommendations.

### Immunoblotting

Splenocytes isolated from PL-treated mice, vehicle-treated EAE mice or control mice were cultured in complete RPMI 1640 medium at a density of 5×10^6^ /ml in the presence or absence of the MOG peptide (20 µg/ml) for 24 h. CD4^+^ T cells were purified using magnetic microbeads (Dynal Mouse CD4 Negative Isolation Kit, Invitrogen, purity >95%). Cells were directly lysed in SDS sample buffer. Proteins were loaded onto a 8% SDS-polyacrylamide gel and subjected to electrophoresis. The separated proteins were electro-transferred onto a nitrocellulose membrane (Schleicher & Schuell MicroScience) using a Mini Trans-Blot apparatus (Bio-Rad). The membrane was then blocked with skimmed milk and probed with specific antibodies (Cell Signaling Technology) followed by data analysis on a Odyssey infrared imaging system (LI-COR).

### Statistics

Student's t test was used to analyze the differences between different groups. Data were presented as mean ± standard deviation (SD). Difference between values was considered statistically significant when p<0.05.

## Results

### Amelioration of EAE by PL treatment

**Figure 1:**
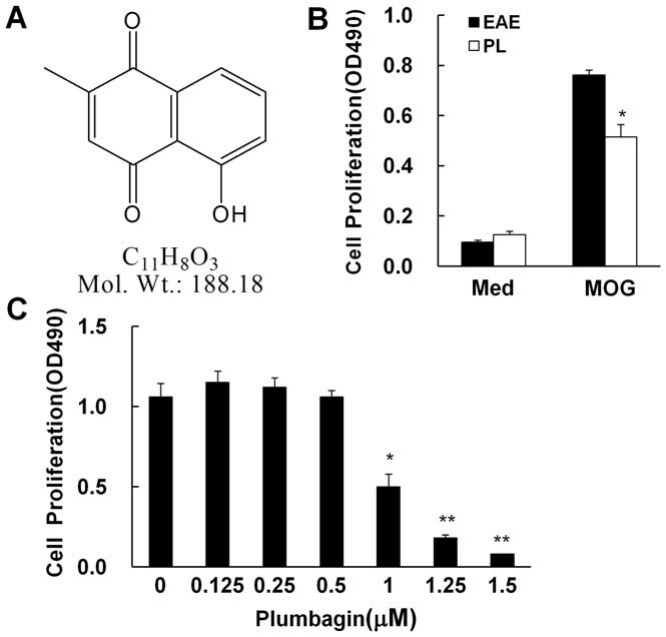
Proliferation of MOG-reactive T cells was significantly inhibited by Plumbagin. A, Chemical structure of Plumbagin. B, Splenocytes were isolated 18 days postimmunization from vehicle-treated and PL-treated EAE mice, and examined *ex vivo* for proliferation by MTS in the presence (MOG) or absence (Med) of MOG peptide. C, Proliferation as measured by MTS in splenocytes from EAE mice stimulated *in vitro* by MOG peptide with or without PL. Data are presented as mean ± SD of triplicates. *p<0.05; **p<0.01.

Splenocytes were isolated from EAE mice and subjected to *in vitro* MOG stimulation. PL, with a structure of 1,4-naphthoquinone (Fig. 1A) was added to test its effect on MOG specific T cell reactivity. It was found that PL significantly inhibit proliferation of MOG-reactive T cells in a dose-dependent manner (Fig. 1C). In addition, PL also significantly inhibited MOG specific T cell reactivity in an *ex vivo* system using splenocytes from vehicle-treated and PL-treated EAE mice stimulated by MOG (Fig. 1B). PL had a low IC_50_ at 1 µM. Under this concentration, it displayed minimum cytotoxicity (data not shown).

**Figure 2:**
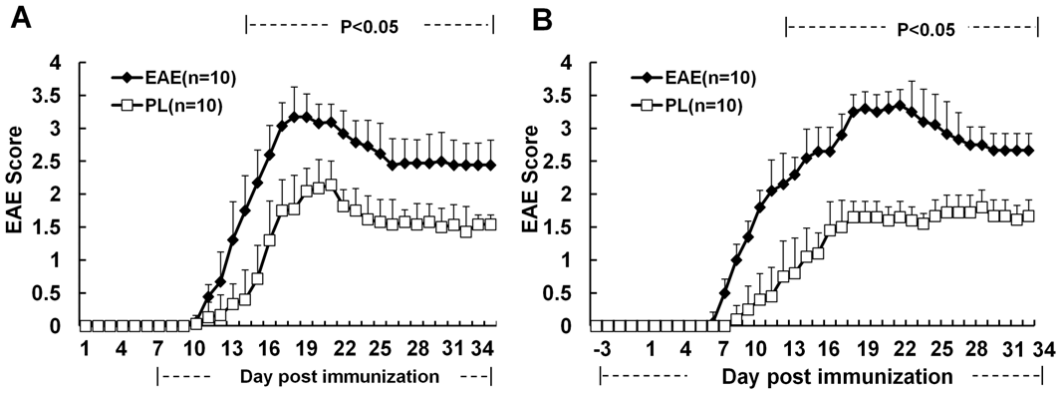
Amelioration of EAE by PL treatment. A&B, Clinical scores of EAE in mice. PL (2 mg/kg) or vehicle control was administered i.p. daily in EAE mice, started from day 7 postimmunization (A) or day3 before immunization (B). Data are expressed as mean ± SD and represent three independent experiments.

**Figure 3:**
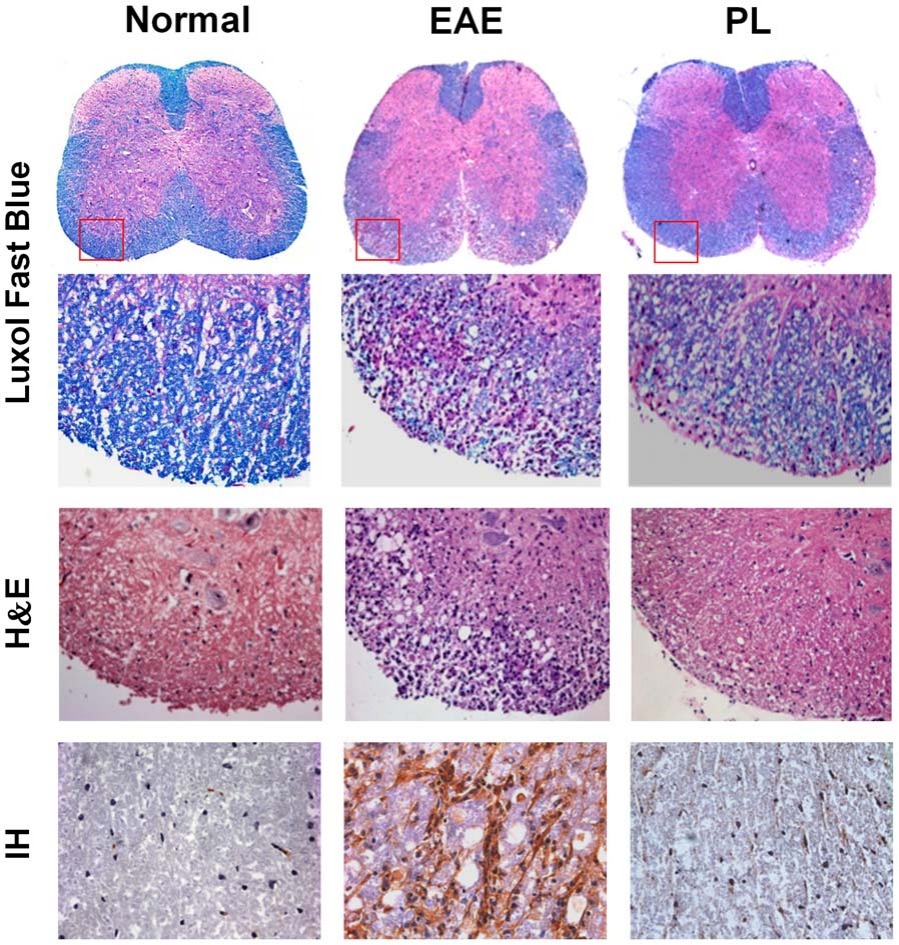
Histology and immunohistochemical staining of spinal cord tissue. Spinal cord sections obtained from normal control mice, EAE mice or PL-treated mice at day 18 postimmunization (treatment protocol) were analyzed by Luxol Fast Blue staining for demyelination, H&E staining for inflammation, and Immunohistochemical staining (IH) for CD4^+^ T cell infiltration. ×25 or ×400. Data presented are representative of three independent experiments. CD4^+^ T cell infiltrates stained by immunohistochemistry were enumerated microscopically in normal mice (10.33±3.51), EAE mice (128.67±7.02) and PL-treated mice (34.33±3.51), respectively. Data are expressed as mean ± SD. p<0.001 for EAE mice versus normal mice, p<0.01 for PL-treated mice versus EAE mice.

As illustrated in Fig. 2, no matter PL was administered at 7 days post immunization, or 3 days before immunization, it resulted in a significant reduction in EAE score as compared with the vehicle control. The observed clinical effect of PL accompanied by a marked decrease of inflammation and demyelination in histological analysis of affected spinal cord. Furthermore, this observation was consistent with the immunohistochemical result which showed a decreased infiltration of CD4^+^ T cells in affected spinal cord of PL-treated mice (Fig. 3). It is evident that the therapeutic effect of PL was associated with a marked reduction in the number of CNS infiltrating encephalitogenic CD4^+^ T cells.

### PL inhibited proinflammatory molecule expression in MOG-reactive T cells

**Figure 4:**
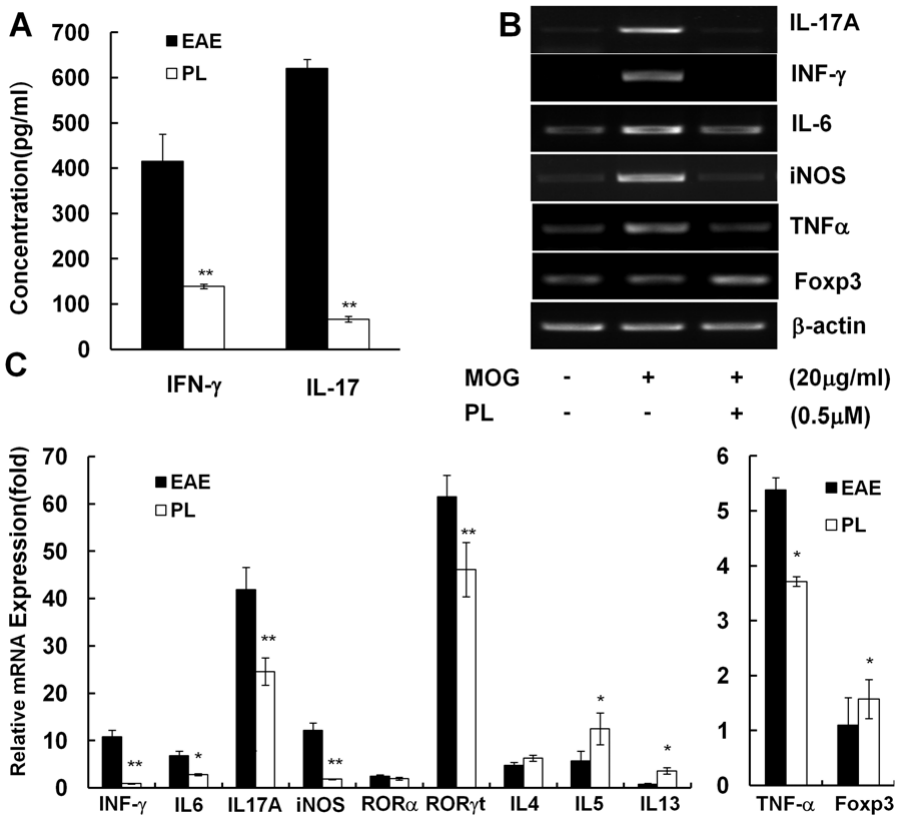
Cytokine and other molecule profiling of MOG-reactive T-cells in response to PL treatment. A, Splenocytes from PL-treated mice (open bars) or EAE mice (solid bars) were challenged with MOG peptide, and culture supernatants were collected at 48 h for cytokine measurement by ELISA. Data are presented as mean ± SD (pg/ml). B, Splenocytes isolated from EAE mice were stimulated with MOG peptide in the presence or absence of PL (0.5 µM) for 24 h. mRNA expression of selected genes was measured by RT-PCR. C, Splenocytes isolated from EAE mice and PL-treated mice were stimulated with MOG peptide for 24 h. mRNA expression of stated genes was measured by real-time PCR.

The significant treatment effect of PL in EAE prompted us to investigate in detail about its potential regulatory mechanisms and to identify the target molecules through which PL might regulate the immune system. To this end, splenocytes were isolated from PL-treated and vehicle-treated EAE mice and characterized for T cell cytokine profile in response to *in vitro* challenge by the disease-eliciting MOG peptide. MOG-reactive T cells derived from PL-treated mice displayed a markedly altered cytokine profile from that of vehicle-treated mice, characterized by significantly reduced production of proinflammatory cytokines including IFN-γ and IL-17 (Fig. 4A). In addition, the effect of PL on mRNA expression of proinflammatory cytokines and other molecules was studied using splenocytes isolated from EAE mice or PL-treated mice and cultured in the presence or absence of the MOG peptide and PL at 37°C in 5% CO_2_ for 24 h. The mRNA expression of IL-17, IFN-γ, IL-6, TNF-α and iNOS were decreased, as well as RORγt expression, which is critically involved in Th17 differentiation. At the same time, it seems that Th2 type cytokines such as IL-5, IL-13 were up-regulated by PL. The mRNA expression of Foxp3, an important transcription factor for Treg cells, was also increased by PL treatment (Fig. 4, B and C).

### The effect of PL on CD4^+^ T cell differentiation was mediated through the JAK/STAT signaling pathway

**Figure 5:**
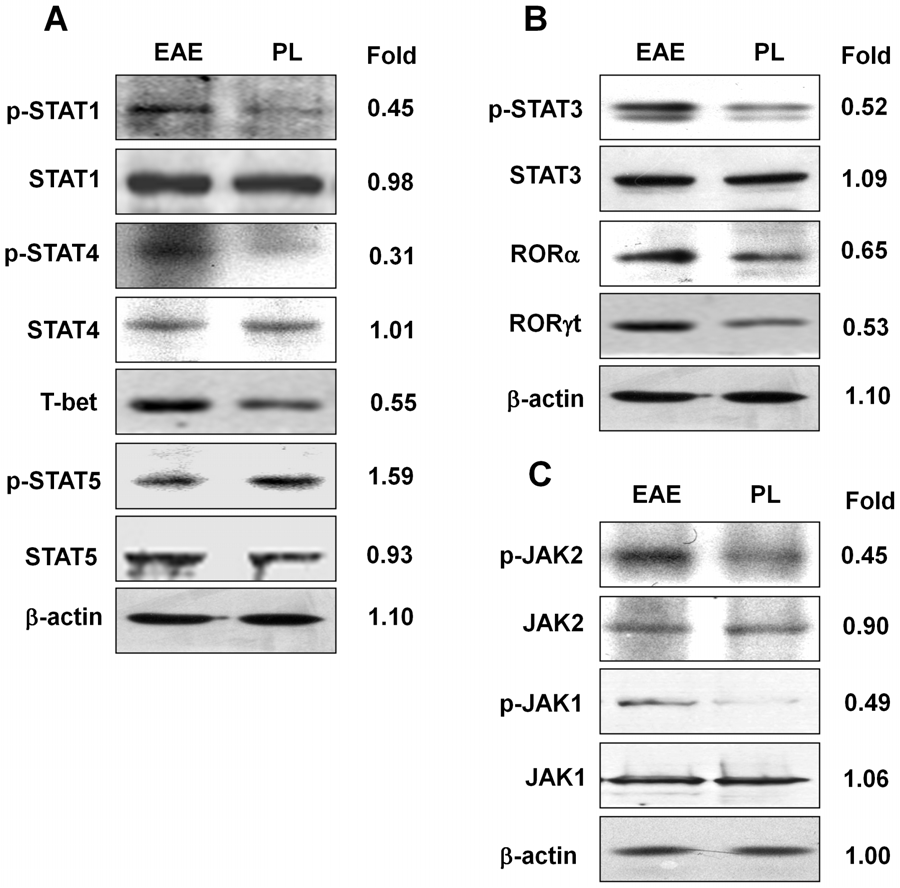
Inhibition of Th1 and Th17 cells by PL through suppressing JAK/STAT pathway. Splenocytes isolated from EAE mice or PL-treated mice were re-stimulated with MOG peptide for 24 h. CD4^+^ T-cells were purified and proteins subjected to electrophoresis and immunoblotting. Membranes were probed with antibodies to JAK, STATs or their phosphorylated form, and other proteins as indicated. A, Molecules related to Th1 differentiation; B, Molecules related to Th17 differentiation; C, JAK family.

Because the JAK/STAT signaling pathway is known to play a critical role in differentiation and survival of Th1 and Th17 lineages, we investigated the expression or phosphorylation level of transcription factors involved in the development of Th1 (STAT1, STAT4 and T-bet ) and Th17 (STAT3, RORγt and RORα). Splenocytes from PL-treated mice, EAE mice or control mice were cultured in the presence of MOG peptide for 24 h. CD4^+^ T cells were purified using magnetic microbeads and subjected to analysis of key signaling molecules of the JAK/STAT pathway. Immunoblotting demonstrated that phosphorylation of JAK/STAT pathway was stimulated by EAE induction (Fig. S1), whereas PL treatment significantly inhibited phosphorylation of STAT1, STAT4 (Fig. 5A) and STAT3 (Fig. 5B) as well as the upstream kinases JAK1, JAK2 (Fig. 5C). The expression of key transcription factors T-bet and RORγt/RORα critically required for Th1 and Th17 differentiation respectively were also markedly inhibited in CD4^+^ T cells derived from PL-treated mice (Fig. 5, A and B). These data provide strong evidence that PL directly inhibited Th1, Th17 differentiation and function.

### Suppressed proinflammatory molecule expression by PL was correlated with the downregulation of NF-κB signaling

**Figure 6:**
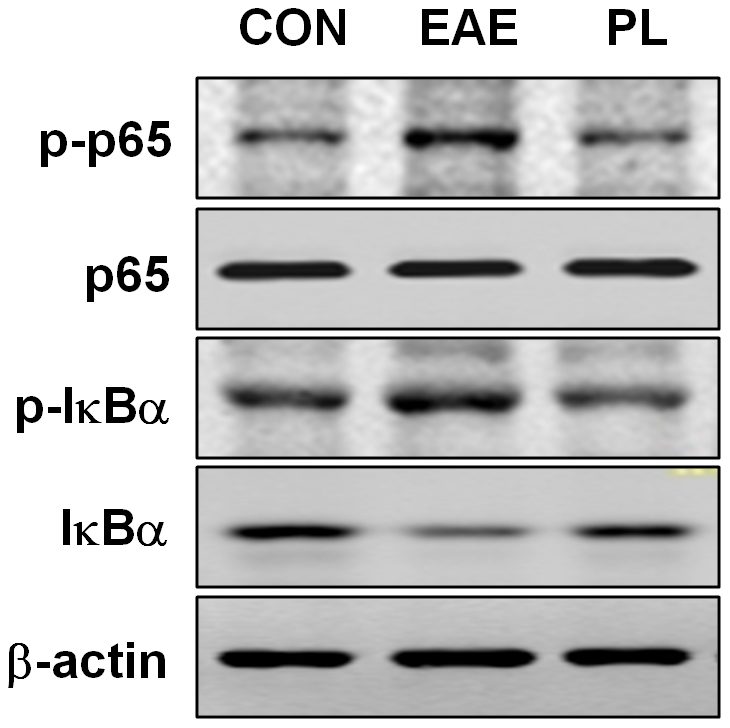
Inhibition of NF-κB signaling pathway by PL. CD4^+^ T-cells from PL-treated mice, EAE mice and adjuvant control mice were cultured in the absence or presence of MOG peptide for 24 h, extracted proteins were subjected to electrophoresis and analyzed by immunoblotting for phosphorylated and total p65 and IκBα.

PL has been shown to suppress the constitutive NF-κB activation in lymphocytes, and the suppression of NF-κB by plumbagin resulted in inhibition of mitogen-induced activation and proliferation of lymphocytes. As we observed the decreased production of proinflammatory molecules such as iNOS, IL-6 and IFN-γ by PL-treated mice (Fig. 4), we proceed to investigate PL's regulatory effects on NF-κB signal pathway in CD4^+^ T cells. As expected, the result revealed that IκBα phosphorylation as well as its degradation was suppressed by plumbagin. In the meantime, the phosphorylation of p65 subunit of NF-κB was also inhibited (Fig. 6).

## Discussion

In inflammatory diseases and autoimmune diseases, CD4^+^ T cells serve as an important source of proinflammatory cytokines. Depending on the environmental signals presented during activation, naive CD4^+^ T cells differentiate into distinct effector lineages [29], [30]. Among these are Th1 and Th17 lineages, imprinted for IFN-γ and IL-17 expression by upregulated expression of T-bet and ROR, respectively. The combination effect of IFN-γ and IL-17 is essential in the effective induction and maintenance of autoimmunity [4], [31], [32].

Although EAE has its drawbacks as a model for human MS, it is still invaluable in investigating basic immunopathological mechanisms of MS, as well as providing a convenient animal model for novel therapy testing and drug mechanism elucidation. Our study presented here demonstrated that PL, a natural bicyclic naphthoquinone, exerts potent anti-inflammatory actions, resulting in amelioration of EAE through unique signaling pathways. PL inhibited MOG-specific lymphocyte proliferation which associates with significant reduction of pro-inflammation cytokines as well as CD4^+^ T cells infiltration into spinal cord tissue. Both treatment and prevention protocols showed that PL can markedly improve the clinical symptom of EAE, but it did not significantly postpone the disease onset. One of the possible explanation is that PL acts directly upon encephalitogenic T cells.

As stated above, Th1 and Th17 cells are main pathogenic T cells critically involved in the disease pathogenesis of MS and EAE model [33], whereas JAK/STAT signaling pathway is one of the major signaling networks that regulate T cell differentiation and their activities [34]. In this study we demonstrated that PL affect pathogenic Th1 and Th17 cells differentiation and functions through regulation of JAK/STAT signaling pathway, resulting in inhibition of pro-inflammatory cytokines such as IFNγ and IL-17. PL treatment inhibited both STAT1 and STAT4 phosphorylation as well as the expression of T-bet in CD4^+^ T cells, altering Th1 differentiation and the clinical course of EAE. It is understood that phosphorylated STAT1 contributes to the transcription of T-bet which bind to IFNγ promoter, drive CD4^+^ T cell differentiate toward Th1 phenotype. Phosphorylated STAT4 also translocates and binds to IFNγ promoter, which initiate IFNγ production and contribute to the full differentiation of a Th1 cell [35]. On the other hand, naïve CD4^+^ T cells differentiation into Th17 cells is mediated by TGF-β and IL-6 signaling via STAT3, RORγt and RORα transcription factors, which is further enhanced and maintained by IL-23 and IL-21[36]. Our *ex vivo* analysis of CD4^+^ T cells from mice immunized with MOG_35–55_ peptide indicated that PL downregulates STAT3 phosphorylation and RORγt/RORα expression in CD4^+^ T cells and decreased the encephalitogenic capacity of these cells. In addition, we found that PL enhanced STAT5 phosphorylation,as well as expression of FOXP3, the important transcription factor for Treg cells, which means that PL might has a role in Treg differentiation. The precise mechanism of the balance between Treg and Th17 differentiation induced by PL still needs to be elucidated [37].

It is worth noticing that NF-κB, a key mediator of inducible transcription in the immune system and a hallmark of inflammatory responses, traditionally focused on its role in the initiation of innate and adaptive immune responses. STAT3 may directly or indirectly interact with NF-κB, as reported previously [38], [39]. Also, PL has been shown to suppress NF-κB activation and NF-κB regulated gene transcription [28]. Consistent with these reports, our results demonstrated that anti-inflammatory effects of PL are likely to involve the NF-κB pathway. PL inhibits MOG induced NF-κB activation in T cells by preventing phosphorylation and degradation of IκBα. Apart from this, plumbagin also inhibited phosphorylation of the p65 subunit of NF-κB. Together, these resulted in the suppression of NF-κB regulated gene transcription including certain proinflammatory cytokines and molecules (e.g. INFγ, IL6, iNOS).

In conclusion, PL exerted the novel anti-inflammatory properties in EAE and resulted in its amelioration. To our knowledge, this is the first demonstration of PL's regulatory effect on T cell differentiation and function, through JAK-STAT pathway. The treatment effect of PL is achieved through targeting multiple signaling molecules critically related to autoimmunity. It raises the possibility that PL may be used as a potential treatment for autoimmune diseases such as MS. In addition, this study provides an example for using natural compounds in probing the complex cytokine signaling network and novel therapeutic targets for autoimmune diseases and other inflammatory conditions.

## Supporting Information

Figure S1 **Phosphorylation of JAK/STAT pathway stimulated by MOG during EAE induction.** Splenocytes isolated from adjuvant control mice, EAE mice and PL-treated mice were re-stimulated with MOG peptide for 24 h. CD4^+^ T-cells were purified and proteins subjected to electrophoresis and immunoblotting. Membranes were probed with antibodies to JAK, STATs or their phosphorylated form. A, STATs, T-bet and ROR; B, JAK family.
(TIF)
