# Supplementary figures and images for: Amelioration of Experimental Autoimmune Encephalomyelitis by Plumbagin through Down-Regulation of JAK-STAT and NF-κB Signaling Pathways
Source: PLoS One. 2011 Oct 31;6(10):e27006. doi: 10.1371/journal.pone.0027006 (PMC3205001; doi:10.1371/journal.pone.0027006)

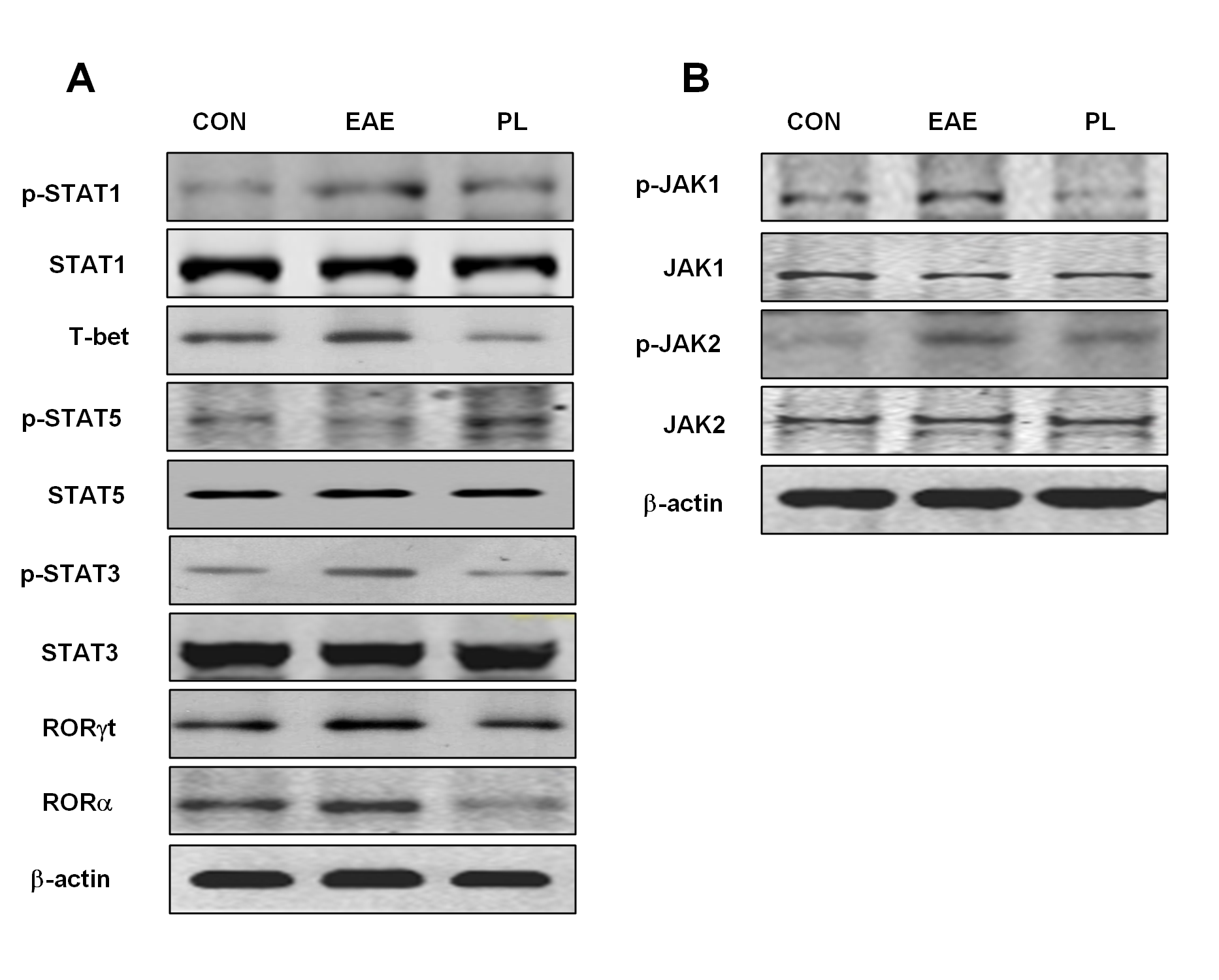

Supplement: Figure S1 — Phosphorylation of JAK/STAT pathway stimulated by MOG during EAE induction. Splenocytes isolated from adjuvant control mice, EAE mice and PL-treated mice were re-stimulated with MOG peptide for 24 h. CD4+ T-cells were purified and proteins subjected to electrophoresis and immunoblotting. Membranes were probed with antibodies to JAK, STATs or their phosphorylated form. A, STATs, T-bet and ROR; B, JAK family. (TIF) [file pone.0027006.s001.tif]
